# Arthritis and Myositis in a Patient Treated with Programmed Cell Death-1 (PD-1) Inhibitor Pembrolizumab for Lung Cancer

**DOI:** 10.31138/mjr.31.3.355

**Published:** 2020-09-30

**Authors:** Eleni Pagkopoulou, Theodora Simopoulou, Eleni Maragkouli, Stamatia Perifanou-Sotiri, Athanasios Kotsakis, Dimitrios P. Bogdanos

**Affiliations:** 1 Department of Rheumatology and Clinical Immunology and; 2 Department of Oncology, University General Hospital of Larissa, School of Health Sciences University of Thessaly, Larissa, Greece

**Keywords:** Immune checkpoint inhibitors, immune related adverse events, myositis, arthritis

## Abstract

Immune checkpoint inhibitors (ICIs) are a new class of drug that have demonstrated efficacy across many cancer types. Because of their nature and mode of action, ICIs unleash immune activation raising concerns as to whether they can be used in patients with concomitant autoimmune or auto-inflammatory diseases. Their usage can lead to the development of autoimmune phenomena known as immune related adverse events (irAEs), virtually affecting every organ. As the use of ICIs is drastically increasing, evidence of irAEs has been accumulating. Herein, we report a case of inflammatory myositis and arthritis 6 months after pembrolizumab therapy, an anti-programmed death-1 (PD1) ICI in a patient with lung cancer, aiming at raising awareness of the diagnostic and clinical challenges clinicians may face when checkpoint inhibitors-related rheumatologic irAEs are developed.

## INTRODUCTION

Immune Checkpoint Inhibitors (ICIs) are a new class of drug that have changed the therapeutic approach for numerous tumours. ICIs are approved for many cancer types, including non-small-cell lung cancer, melanoma, and renal cancer. They exhibit antitumour effects by blocking the action of immune inhibitor signalled cytotoxic proteins, such as cytotoxic T-lymphocyte-associated protein 4 (CTLA-4), programmed cell death protein 1 (PD-1), and programmed cell death ligand 1 (PD-L1).^1^

Pembrolizumab is a humanized monoclonal IgG4 isotype antibody initially approved as monotherapy for the treatment of metastatic melanoma and refractory NSCLC.^2^ PD-1 is a molecule expressed on the surface of antigen stimulated T-cells, but also in macrophages, B cells, natural killer T cells, and dendritic cells.^3^ Pembrolizumab targets the PD-1 and blocks interaction between PD-1 and its ligands, PD-ligand 1 (PD-L1) and PD-ligand 2 (PD-L2).^2^ Under normal conditions, PD-1 and related PD-L1 are expressed on the surface of activated T cells. PD-L1/PD-1 interaction inhibits immune activation and reduces T-cell cytotoxic activity when bound, an essential process for maintaining normal immune responses. Tumour cells make use of the PD-1 pathway to circumvent T-cell–mediated cytotoxicity by expressing PD-L1 on the tumour itself or on tumour-infiltrating immune cells. This may lead to inhibition of immune-mediated death of tumour cells permitting cancer cells to grow and spread.^4^

Immune activation caused by this therapeutic blockade is causing immune related adverse events (irAEs). In a single, open-label, multicentre, Phase I trial study in patients with progressive, locally advanced or metastatic carcinoma, melanoma and non-small cell lung cancer, about 13% of patients in the pembrolizumab group developed irAEs, such as thyroid disorders, pneumonitis and colitis which can be severe (grade III, IV or V) in 5–10%.^5^ Among irAEs, rheumatic and musculoskeletal events have to date not been widely characterized; arthralgia and myalgia are common AEs, whereas arthritis, myositis and vasculitis have been reported in case series and case reports.^6^ Case reports reveal a diverse group of rheumatic conditions after treatment with these agents that require referral to rheumatologists.

In this report we present a patient who developed immune-mediated myositis and arthritis 6 months after pembrolizumab was administered for lung cancer.

## CASE REPORT

A 64-year-old Caucasian male, without pre-existing autoimmune disease, was diagnosed with adenocarcinoma of the right lung on September of 2017, whose immunohistochemical staining showed neoplasm with increased expression of PD-L1. The oncologists decided to treat him with pembrolizumab. After 5 months he developed deep vein thrombosis (DVT) in the left lower limb, and 2 weeks after, he also developed arthritis of the right knee. Progressively he reported arthritis of the left knee as well, along with decreased muscle strength of the lower limbs. He was referred to our Rheumatology Unit; at presentation, arthritis of both knees was confirmed along with decreased muscle strength of the iliopsoas and quadriceps muscles. The rest of clinical evaluation was unremarkable.

Laboratory workup was performed. The patient had increased inflammation markers ESR 73 mm (<15) and CRP 4mg/dl (< 0.5mg/dl). Complete blood count was unremarkable as well as biochemical tests, with the exception of a slight increase in creatine kinase (CPK) levels (CPK 227IU/L, <200); alanine aminotransferase (ALT), aspartate aminotransferase (AST) and aldolase were within normal range. Electromyography revealed spontaneous activity and polyphasic potentials of short duration and low amplitude, compatible with inflammatory myopathy. The patient was seronegative for rheumatoid factor (RF) and anti-cyclic citrullinated peptide (CCP) antibodies. Antinuclear antibodies were positive at 1:160 titre with speckled pattern, but anti-Jo1 antibodies were negative. Further testing for antigen-specific autoantibodies related to inflammatory myopathies by line immunoassay (Euroimmun, Lübeck, Germany) has turned negative for Mi-2 alpha, Mi-2 beta, TIF1g, MDA5, NXP2, SAE1, Ku, PM-Scl100, PM-Scl75, Jo-1, SRP, PL-7, PL-12, EJ, OJ, Ro-52. Muscle biopsy was not performed as the patient was on new oral coagulant (rivaroxaban) due to a recent deep venous thrombosis episode (2 months before his admission) and refused any surgical procedures. The patient underwent an imaging scanning with lung and abdominal CT, without evidence of cancer recurrence.

Taking into account clinically evident muscle weakness, as well as the EMG findings and the elevated inflammatory markers, regardless of the nearly normal CPK, the diagnosis of inflammatory myopathy along with knee arthritis was made and the patient received steroids (prednisone 20mg/day) and methotrexate 10mg/week. A rapid and significant clinical improvement of his arthritis and muscle weakness was observed. Twelve months after diagnosis, prednisone was gradually tapered and discontinued along with the methotrexate and the patient is in good clinical condition, with normal muscle strength and without swollen and/or tender joints.

## DISCUSSION

Immune-checkpoint inhibitors represent a novel therapeutic approach for many malignancies. They block the negative interactions between T-cells, antigen presenting cells, and cancer cells, allowing T-cell activation. Their therapeutic blockade can alter immunological tolerance, which results in immune-related adverse events.^1^ IrAEs have affected nearly every organ system and range widely in severity. While colitis, hepatitis, and pneumonitis are well documented, rheumatic and musculoskeletal involvement is less described and appreciated.^7^

The mechanisms responsible for ICI-induced irAEs remain under investigation. Possible pathogenetic mechanisms include the increase in T-cell activity against neoantigens presented in both tumour and healthy tissues, the increase in pre-existing antigen-specific autoanti-body levels, bearing pathogenic potential, the increase in levels of pro-inflammatory cytokines, and increased inflammation mediated by the complement via the direct binding of anti-CTLA4 antibody, with the CTLA-4 receptor expressed in normal tissue.

To our knowledge, irAEs from pembrolizumab are largely restricted to case study reports.

Arthralgia is among the most commonly reported AEs in clinical trials.^5,6^ The incidence of arthralgia is around 9–12% for patients receiving pembrolizumab, whereas frank inflammatory arthritis is rarely reported.^6^ It may present with different clinical phenotypes; monoarthritis, oligoarthritis, or as acute and severe polyarthritis, with male sex predominance.^8^ Most authors report seronegative arthritis, while anti-CCP positive rheumatoid arthritis has also been reported.^8^ The differential diagnosis of septic arthritis or metastasis should be a priority in all cases of persistent arthritis.

Myalgia was the second most commonly reported musculoskeletal complaint in clinical trials, sometimes with severe fatigue resembling polymyalgia rheumatica.^6^ True inflammatory myositis, with clinically evident muscle weakness, has rarely been reported. It should be mentioned that it can present as reactivation of a pre-existing paraneoplastic polymyositis and dermatomyositis or as a new onset myositis. Immune-mediated neuromuscular side-effects of checkpoint inhibitors vary in presentation and differ from their idiopathic counterparts.^9^ Patients may present with dermatomyositis; however, atypical presentations also occur. Fulminant myositis may have a severe course, affects vital muscles such as the myocardium, and requires urgent treatment. Polyneuropathy, myasthenia gravis and myositis coexist in some patients receiving ICI causing problems involving all neurological muscular junctions.^9^ Instrumental findings may differ from typical neuromuscular disorders occurring outside ICIs treatment, making differential diagnosis difficult.

Regarding therapy, systemic corticosteroids have been administered in most cases of arthritis, while NSAIDs, hydroxychloroquine or methotrexate have also been used.^6^ Systemic corticosteroids are the treatment of choice also in myositis.^6^ The efficacy of other immunosuppressants and intravenous immunoglobulin (IVIG) that are commonly used in dermatomyositis, has not been clearly assessed. In 2018 the American Society of Clinical Oncology set some recommendations regarding the management of irAEs in patients treated with ICI therapy.^10^ They intend to provide an initial guidance to treatment and also underline the need of referral to other specialties, like the rheumatologists. For inflammatory arthritis, paracetamol, NSAIDs, prednisone, and/or intra-articular corticosteroids are the first step, while for more severe cases, or if no improvement occurs after 4–6 weeks, then synthetic and biologic DMARDs should be considered. According to the grade of the myositis, acetaminophen and/or NSAIDs and prednisone at 0.5 to 1 mg/kg, or other immunosuppressive therapy, such as methotrexate, azathioprine, or mycophenolate mofetil, is recommended. In more severe cases plasma exchange, IVIG therapy should be considered. More recently, the European League Against Rheumatism (EULAR) has issued recommendations for diagnosing and managing rheumatic-related adverse events associated with cancer immunotherapy. They include treatment escalation strategies, including local/systemic corticosteroids, conventional synthetic DMARDs, and biological DMARDs for severe or refractory irAEs along with a special warning on severe myositis.^11^

Immune checkpoint inhibitors have shown promising results in neoplastic disease, but their use can be hindered by serious immune-related adverse events. Proper management of autoimmune adverse events and indeed full-blown autoimmune rheumatic disease require collaboration between oncologists and rheumatologists for prompt recognition of the rheumatic disease and treatment. Since ICIs are increasingly being used, clinical awareness on immune-related adverse events should be increased.
